# Analysis of Mass Transfer and Shrinkage Characteristics of Chinese Cabbage (*Brassica rapa* L. ssp. *pekinensis*) Leaves during Osmotic Dehydration

**DOI:** 10.3390/foods13020332

**Published:** 2024-01-20

**Authors:** Timilehin Martins Oyinloye, Won Byong Yoon

**Affiliations:** 1 Department of Food Science and Biotechnology, College of Agriculture and Life Sciences, Kangwon National University, Chuncheon 24341, Republic of Korea; oyinloyetm@kangwon.ac.kr; 2 Elder-Friendly Research Center, Agriculture and Life Science Research Institute, Kangwon National University, Chuncheon 24341, Republic of Korea

**Keywords:** Chinese cabbage, diffusion, shrinkage, deformation, mass transfer model, numerical simulation

## Abstract

**Highlights:**

**What are the main findings?**

- The diffusion coefficient of the Chinese cabbage leaf is insignificantly different regardless of its position due to similarities in leaf thickness.
- Soluble solid uptake by the Chinese cabbage midrib is not influenced by shrinkage for a brining period between 0 and 48 h.
- More rapid soluble solid uptake occurs in the Chinese cabbage leaf blade than in the midrib.
- Numerical models provide insight into the influence of shrinkage during Chinese cabbage osmotic dehydration.

**What is the implication of the main finding?**

- Optimal Brining Conditions: Different sections of Chinese cabbage (CC) respond uniquely to brining time, suggesting the need to optimize brining conditions based on the equilibrium concentrations observed at positions 1 to 4.
- Early Soluble Solid Uptake: Rapid soluble solid uptake in CC leaf blade and midrib during the initial 0–18 h underscores the importance of monitoring and controlling the early stages of brining to influence efficient soluble solid uptake.
- Shrinkage Effect Considerations: Shrinkage significantly affects CC midrib in the first 18 h and becomes visible between 48 and 120 h, emphasizing the time-dependent impact of shrinkage. For short-term brining (up to 48 h), shrinkage may be negligible, but for longer durations, especially midrib at position 1, considering shrinkage is crucial for accurate modeling.
- Computational Simplification: Experimental and simulated results agreement suggests that, for short-term brining processes, excluding the shrinkage effect from diffusion models may be justifiable. This implies a potential reduction in computational complexity without compromising accuracy.

**Abstract:**

The mass transfer and shrinkage characteristics of Chinese cabbage (CC) during osmotic dehydration (OD) were investigated. The leaves were grouped into four sections and analyzed based on their morphological characteristics (i.e., maturity, width, and thickness). The sections were immersed in 2.0 mol/m^3^ NaCl for 120 h at 25 ± 2 °C. The diffusion coefficient (D) of the leaf blade was not significantly different with respect to the sections that were formed, but it was significantly different in the midrib in the increasing order of P1, P4, P3, and P2, with values of 1.12, 1.61, 1.84, and 2.06 (× 10^−6^), respectively, after a 1 h soaking period due to the different characteristics in morphology and structure, such as porosity (0.31, 0.41, 0.42, and 0.38 for positions 1, 2, 3, and 4, respectively) and fiber contents. Numerical simulation (NS) for CC was conducted with and without the consideration of shrinkage during OD. The shrinkage effect on the NaCl uptake analyzed using NS indicated no significant difference between 0 to 48 h for both models. However, changes in the NaCl concentration were observed from 48 h onwards, with a lesser concentration in the model with shrinkage for all sections. The difference in NaCl concentration for the models with and without shrinkage was within the standard error range (±0.2 mol/m^3^) observed during experimental analysis. This implies that the shrinkage effect can be overlooked during the modeling of CC to reduce computational power.

## 1. Introduction

Chinese cabbage (CC) (*Brassica rapa* L. ssp. *pekinensis*) is a popular leaf vegetable that has been grown in eastern Asia for many centuries [1]. In Korea, CC is the primary raw material ingredient in *Kimchi*, a salted and fermented dish [2]. An important processing step that determines the texture, chemical, and physical properties of *Kimchi* is the salting process, often referred to as osmotic dehydration (OD) [3].

OD is a solid-to-liquid contact operation involving the immersion of food in hypertonic solutions [4]. The difference in the osmotic pressure between the food and the solution results in the diffusion of water from the food into the solution and the diffusion of the solute from the solution into the food [5]. OD has been considered an effective pretreatment that contributes to improving the final product’s quality characteristics and extending the shelf life [6,7]. However, OD causes several changes in food, and one of the most noticeable is product shrinkage [8,9].

The effect of the mass transfer of the solute into cabbage during OD has been widely studied [10,11]. However, most studies analyzing OD in cabbage consider only the variable effective diffusivity without considering the morphological properties of the leaves, and product shrinkage is frequently overlooked [12,13]. According to da Silva et al., [14], assuming the change in effective diffusivity without accounting for the shrinkage may result in a significant inaccuracy during OD. This is because shrinkage modifies the internal structure of the product, thus altering the effective diffusivity. Recently, a few studies have analyzed OD alongside the shrinkage effect in fruits and vegetables. Aguirre-Garcia et al., [15] studied mass transfer during OD in papaya cubes immersed in a sucrose solution and found that OD analysis without considering the shrinking resulted in a significant overestimation of the diffusivity. Similarly, Zecchi and Gerla [16] studied the OD of sliced tomatoes. The authors compared rigid solid and shrinkage models and reported that considering the shrinkage factor in tomatoes resulted in a lower diffusion coefficient than in rigid solids. However, materials behave differently under osmotic conditions, and because there are no models that incorporate this feature in CC, it is unknown how the interacting nature of the dehydration and impregnating processes will affect both the water loss and soluble solid gain behavior, as well as the mass transfer property estimation.

In addition, CC has distinct leaf and stem regions with significantly different structures, thus making modeling of the OD in CC extremely complex [17,18]. No study was found to have considered OD and the influence of shrinkage in CC; this may be due to the complicated structure. For many other types of fruits and vegetables with a complexity in morphology, many authors have resolved to use simplified models [18,19,20]. This is because most foods are considered to be a porous medium, and the analysis of mass transfer through porous media is a computationally intensive task requiring information about the pores’ topology, flow paths, and liquid–solid interactions. Therefore, several modeling strategies with different simplification degrees have arisen when describing the OD operation, including modification of the food shape to eliminate parts that may prolong the modeling process and the assumption that food exists as a two-dimensional object. For example, González-Pérez et al., [20] developed a 2D mass transfer shrinkage model to estimate the water and solute diffusivity in mushrooms using simplified models developed from images acquired from slices of mushrooms. However, to fully understand and characterize the mass transfer process and shrinking effect during OD, selecting an appropriate shape and size to represent the product is a critical factor in determining the model’s reliability. Thus, one must explore a technique that adequately captures the product’s form and size without excess model simplification, particularly as a 3D spatial domain. Thus, this research addresses this major gap in the literature, since prior studies have mostly concentrated on the mass transfer of the solute during osmotic dehydration (OD) in cabbage, thus ignoring the morphological features and shrinkage consequences. In contrast to traditional evaluations that only take into account effective diffusivity without taking into account the complex leaf structures, this study also incorporates CC morphological traits into the modeling procedure, thus providing information on the intricate interactions that occur during OD between mass transfer characteristics, soluble solid gain, and water loss.

Therefore, this study aimed to evaluate shrinkage’s effect on the soluble solids (NaCl) gain in the nonsimplified shape of CC during the salting process. CC leaves were divided into sections based on morphological characteristics to describe the model effectively, and a salting process was conducted on separated leaves. This will serve as a foundational study to develop diffusion and shrinkage models for a whole cabbage with accurate model geometries.

## 2. Materials and Methods

### 2.1. Sample Preparation

**Figure 1:**
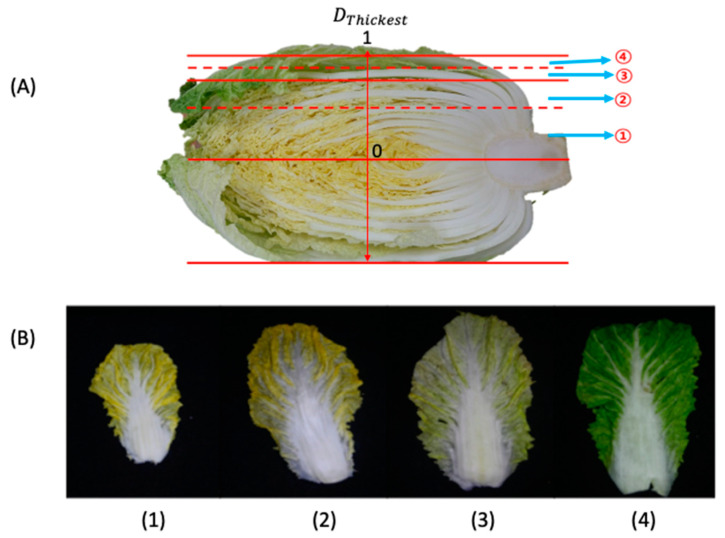
Schematic representation of different positions and division method of Chinese cabbage leaves (**A**) and separated leaves from the identified positions (**B**). Numbers 1, 2, 3 and 4 denote the leaves in positions 1, 2, 3, and 4, respectively.

Fresh CC was purchased from a local market (Chuncheon, Republic of Korea). The initial CC weight and moisture content for the leaves and stem measured following the AOAC [21] method were 0.468 ± 0.11 kg and 91.73 ± 1.28% for leaves, and 1.372 ± 0.32 kg and 93.65 ± 2.28% for stem. A dimensionless number was created to divide the leaves into four different categories, because the sizes and quantities of leaves in a bunch of CC varied. Since the leaves grow horizontally, CC was first divided into two halves along the horizontal axis, and a part of the two halves was subsequently divided into four distinct categories (Figure 1A).

**Figure 2:**
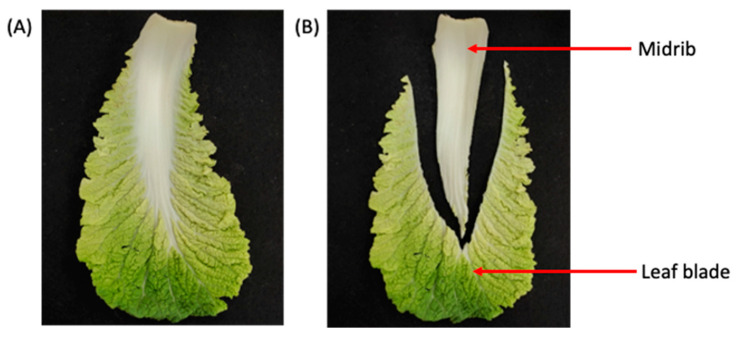
Schematic representation of Chinese cabbage leaf: (**A**) full image of leaf and (**B**) image of separated leaf into midrib and leaf blade.

As shown in Figure 1A, the dimensionless number was measured along the thickest region on the half cabbage flat surface, i.e., the area marked as D_Thickest_. From the midpoint along the D_thickest_ area, the length was measured and represented as a dimensionless numbers between 0 to 1, with 0 being the center of the half CC, and 1 being the outer edge along the D_thickest_ area (Figure 1A). Four distinct groups were created based on the dimensionless number as follows: position 1 (0 to 0.5), position 2 (>0.5 to 0.75), position 3 (>0.75 to 0.875), and position 4 (>0.875 to 1). The dimensionless number developed resulted in the grouping of the CC leaves based on their developmental stage, with position 1 being the smallest and the youngest leaves being located in the center of the CC. Position 4 contained the largest, oldest leaves and was located in the most exterior parts (Figure 1B). In addition to the CC leaf categories created, the CC leaves were further divided into the midrib and leaf blade sections (Figure 2). The analysis conducted in this study was carried out on the different parts identified in Figure 2.

### 2.2. Brining Process

**Figure 3:**
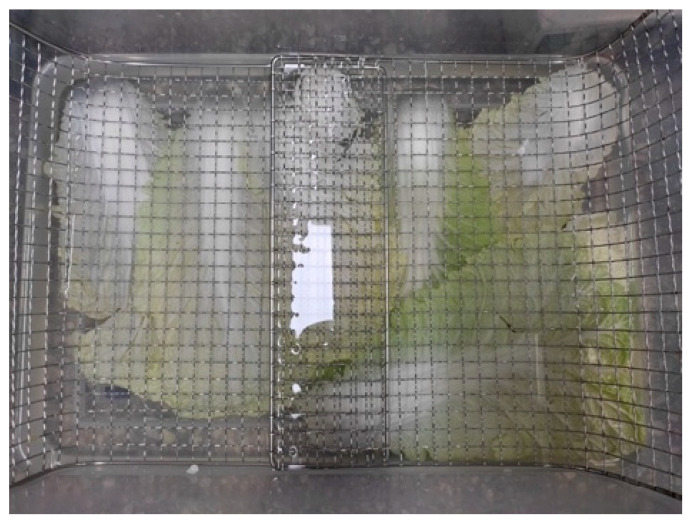
Container with 8 Chinese cabbage leaves in the brine solution, with 2 leaves representing one position.

Samples of CC leaves from positions 1 to 4 were collected, and each leaf was immersed in an NaCl solution of 2.0 mol/m^3^. The concentration of the brine solution used in this study was based on expert recommendation following the concentration level used in the commercial production of *Kimchi* (Korea Food Research Institute, Seongnam-si, Republic of Korea). To achieve a concentration of 2.0 mol/m^3^, 2045 g of premium table salt (Young Jin Green Foods Co., Ltd., Gangnam-gu, Seoul, Republic of Korea) was dissolved in 12.955 L of distilled water. A wire mesh was placed over the brine solution to avoid buoyancy (Figure 3). The ratio of CC leaves to brine solution was 1:10 by weight. The operating temperature was maintained at room temperature (25 ± 2 °C). Sampling intervals (1, 2, 3, 5, 8, 12, 17, 24, 48, 53, and 120 h) were randomly selected to analyze salt concentration and volume shrinkage. Initial concentrations were determined at the start of the brining process, and the equilibrium concentration of soluble solids (NaCl) was determined as the period when changes in the soluble solid concentration in the CC leaf were no longer detected. The total time (120 h) was more than the time required for the CC leaves to reach equilibrium concentration. At the end of each time interval, the CC leaves were washed with clean running water before analysis, and each batch was discarded once a leaf was removed from it at the evaluation time.

### 2.3. Shrinkage Analysis

The volume of CC before and after dehydration was determined using a liquid displacement method [22]. Following the completion of a given brining time, a weighed mass of CC midrib or leaf blade was placed in a measuring cylinder containing a known volume of ethanol at room temperature, and the new volume was recorded. The shrinkage was defined as the volume ratio (VR) of the osmotic dehydrated sample volume to the original sample volume.

(1) $$Shrinkage\left(\%\right)=VR=\frac{V_{t}}{V_{0}},$$

where V_0_ and V_t_ are the initial and osmotic dehydrated sample volumes, respectively. All measurements were performed five times.

### 2.4. Porosity Measurement

Vacuum impregnation was used to assess the porosity of the CC midrib. The term impregnation refers to the process of being filled [23]. A 40 mm length of CC midrib with an average weight of 0.0086 ± 0.001 kg was submerged in distilled water and exposed to vacuum pressure. The operation continued until there was no buoyancy in the midrib. The sample’s pour volume (V_p_) was calculated using Equation (2):

(2) $$V_{p}=\frac{{W_{saturated}-W}_{i}}{\rho_{w}},$$

where W_saturated_ is the weight (kg) of the sample after vacuum application, W_i_ is the initial weight, and ρ_w_ is the density of water [23,24]. Afterward, the porosity (φ) of the midrib was calculated using Equation (3):

(3) $$\phi =\frac{V_{p}}{V_{i}},$$

where V_i_ is the initial midrib volume.

### 2.5. Prediction of Soluble Solid Concentration

#### 2.5.1. Modeling of Mass Transfer

This study considered soluble solids (NaCl) as a single pseudocomponent in both the solid (CC) and the osmotic solution. As the two components that are transferred between the solid phase and the osmotic solution are water and soluble solids, this system is regarded as pseudobinary in the field of mass transfer. Similarly, the brining process can be modeled as a pseudobinary system, because the essential variables are water and soluble solids concentration [16]. A phenomenological model was formulated considering diffusive control in a homogeneous and isotropic solid whose volume changes during the OD process. Mass transfer during this process can be described using the following mass balance Equation (4) [16,25]:

(4) $$\frac{{\partial (Vc}_{i})}{\partial t}=\nabla (VD\nabla c_{i})-A_{m}J_{i},$$

where V is the sample volume, subscript i represents solvent or solutes, c_i_ represents the mass concentration of component i (soluble solids or water), t is the time, D is the diffusivity, A_m_ is the interface area between the sample and the solution, and J_i_ is the molar flux of component i from the CC volume into the surrounding volume. A model that accounts for both volume change and diffusion in the solid was used to analyze the impact of CC shrinkage throughout the brining process [16]. Due to shrinkage, a new variable ($c_{i}^{o}$), which is defined by Equation (5), was introduced to consider the effect of moving interface.

(5) $$c_{i}^{o}=c_{i}\frac{V_{t}}{V_{0}},$$

where $c_{i}^{o}$ is the concentration of component i during shrinkage, and V_t_ and V_0_ refer to the sample volume at time t and the initial volume, respectively. This means that the mass concentration (c_i_) of each component at time (t) in Equation (4) is modified by the ratio of solid volume at time t and initial sample volume (V_t_/V_0_), thereby allowing to factor the shrinkage of solid during the process. Some simplifying assumptions were also made during the analysis; these include the following [16]:

- The leaf blade has no shrinkage effect due to its relatively small thickness (0.7 mm);
- The solid material is considered porous and isotropic;
- The temperature of the solid and osmotic solution remains constant throughout the process;
- No flux condition at the boundary of the brining tank exists;
- There is no chemical reaction in the system.

#### 2.5.2. Calculation of Salt Concentration in Chinese cabbage

The salt concentration in CC was determined through the titration method described by Korkmaz [26]. The midrib and leaf blade of CC were each blended with distilled water at a ratio of 1:10 by weight using an electric blender (Ultrablend BL935, Tefal S.A.S, Rumilly, Haute-Savoie, Rumilly, France). Before titration, 1 mL of a 50 g L^−1^ potassium chromate solution was added to the blended sample and vigorously shaken. Subsequently, 20 mL of the blended sample was titrated with 0.1 mol L^−1^ silver nitrate solution until the mixture exhibited a reddish-brown color endpoint. The obtained result was expressed as the mass/volume concentration of sodium chloride (g L^−1^), which was calculated using Equation (6):

(6) $$C=\frac{0.05844\times C_{2}\times (V_{2}-V_{1})\times K}{V_{ds}}\times 1000,$$

where 0.05844 represents the mass (g) of sodium chloride equivalent to 1 mL of the silver nitrate standard solution with a concentration of c(AgNO_3_) = 1.000 mol L^−1^. C_2_ (mol L^−1^) denotes the concentration of the silver nitrate standard solution, V_2_ – V_1_ (mL) represents the volume of the silver nitrate solution utilized for titration, K denotes the dilution factor, and V_ds_ (mL) is the volume of the diluted sample.

#### 2.5.3. Diffusivity Analysis

The quantitative analysis of the transfer of soluble solids between the CC and the surrounding solution during OD depends on the diffusion coefficient (D). The D was modeled based on an analytical solution of Fick’s second law described by Equation (7).

(7) $$\frac{C_{0}-C}{C_{0}-C_{eq}}=1-\frac{8}{\pi^{2}}\sum_{n=0}^{\infty }\frac{1}{{\left(2n+1\right)}^{2}}exp\left(\frac{{-D(2n+1)}^{2}\pi^{2}t}{L^{2}}\right),$$

where C is the NaCl concentration at time t, C_0_ is the salt concentration in the CC before the brining process, C_eq_ is the equilibrium concentration of salt in CC, and L is the CC midrib or leaf blade thickness. Because the volume of the CC is continuously changing with brining time, it is assumed that the material properties will change, which may influence the value of D. Therefore, the curve fitting tool in MATLAB (Mathworks^®^ Inc., Natick, MA, USA) was used to analyze the D as a function of the leaf blade and midrib soluble solid concentration with respect to the evaluation time. This gave an estimated diffusion coefficient. However, there are great truncation errors, especially for short brining periods. To avoid the great truncation error, a trial-and-error method, as described by Wang et al. [27], was used in this study to calculate the diffusivity by summing up the first 10 terms on the right side of Equation (7). The calculation strategy resulted in the D value, which was used for the numerical analysis.

#### 2.5.4. Geometry Model

**Figure 4:**
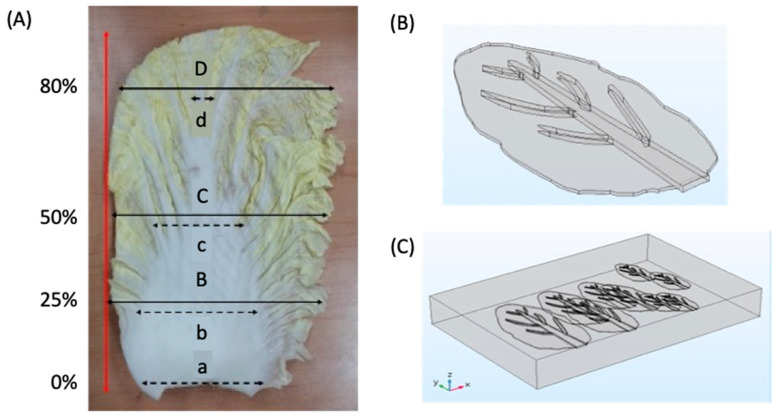
Morphological description of Chinese cabbage leaves (**A**) and a description of a geometrical model for the simulation of cabbage leaves during the brining process (**B**,**C**).

To develop a precise geometry model that represents the average morphology of each group of the CC, the midrib and the leaf blade were separated and measured as described in Figure 4. Based on the overall leaf length, the width of the CC was measured at four consistent locations on the leaves (i.e., position at 0, 25, 50, and 80% of the leaf length). As shown in Figure 4A, the upper-case letters (B–D) represent the width of the leaf blade at each position, while the lower-case letters (a–d) represent the width of the midrib at each position. The average value obtained from the measurement was used to design the geometry model for the CC as shown in Figure 4B, and the leaves representing each position 1, 2, 3, and 4 were arranged as shown in Figure 4C. This arrangement is similar to the one used during the brining process.

#### 2.5.5. Model Implementation

The governing equations for mass transfer phenomena and shrinkage in CC were solved using the finite element method (FEM) using COMSOL Multiphysics® (version 6.0, COMSOL Inc., Burling, MA, USA). A sizing and free tetrahedral function was used to generate a mesh with 33,326 elements. The physics model utilized includes transport diluted species and deformed geometry. This model considers the mass transfer through cellular membranes and the diffusion of different species through intercellular spaces based on the microstructural properties of the CC leaves, as well as the influence of the shrinkage. The model for CC shrinkage was applied to the faces of the midrib by defining the volume shrinkage ratio. In contrast, the side of the midrib was assigned as free deformation with respect to the midrib face due to its relatively small size. During the shrinkage analysis, the mesh quality of the model decreased, which caused difficulty in the convergence of the solution. Therefore, an automatic mesh was applied when the mesh aspect ratio quality dropped below 0.5. This condition pauses the solution and generates a new mesh based on the last solution and continues the simulation process afterwards.

### 2.6. Statistical Analysis

At least five CC leaves were characterized for positions 1, 2, 3, and 4. Mean values and standard deviations were calculated for each measurement of volume shrinkage and soluble solid uptake. The data were analyzed using the SPSS 22.0 statistical analysis program, and the results were reported as mean values ± standard deviations. To estimate the quality of the model developed, the average relative error (ARE) (Equation (8)) was used:

(8) $${ARE}_{ss}=\sum_{i}\left|\frac{C_{ss}^{exp}-C_{ss}^{cal}}{C_{ss}^{exp}}\right|$$

where the subscript ss indicates soluble solids in CC; the superscript exp represents the experimental data; the superscript cal refers to the values calculated from the model; and the counter i indicates that the sum is made for discrete time steps in which experiment data are available.

## 3. Results

### 3.1. Morphological Description of the Chinese Cabbage leaves

The morphological description of the CC leaves is shown in Table 1. The average thickness of the leaf blade was 0.7 ± 0.1 mm and was consistent across all positions, whereas the thickness of the midrib was 6.8 ± 0.2, 7.1 ± 0.1, 7.5 ± 0.1, and 7.4 ± 0.1 mm for positions 1, 2, 3 and 4, respectively. The length of the CC increased significantly from position 1 to 4. This implies that the CC leaves grew longer as they matured. This is consistent with the findings of Gil et al. [28], who identified cabbage vegetable length as an indicator of its maturity, and a fully matured leave can range from 90 mm to > 200 mm. The average length and width values obtained in each position were used to develop the 3D geometry model for numerical simulation.

### 3.2. Equilibrium Concentration, Diffusion Coefficient, and Porosity of Chinese Cabbage

The equilibrium concentration and the time required to attain equilibrium in the CC leaf blade and midrib immersed in a NaCl concentration of 2.0 mol/m^3^ are shown in Table 2. The equilibrium concentrations of the leaf blade for positions 1 to 3 did not differ from one another. However, in contrast to positions 1 to 3, position 4 was different and significantly higher. This could be because of the varying morphological characteristics of the leaf blade, such as their maturation stage, width, and length [28]. As discussed in Section 3.1, the thickness of the leaf blade was not significantly different (0.7 ± 0.1 mm). However, the width and length of the leaves increased from positions 1 to 4. In addition, the width of the leaf blade at position 1 to 3 was about 50% of the total CC width, whereas the width of the leaf blade in position 4 occupied about 60 to 70% of the CC leaves. Thus, the equilibrium concentrations of the leaf blades in positions 1 to 3 were not significantly different. On the other hand, the leaf blades in position 4 were more mature than the leaves in positions 1 to 3; hence, the equilibrium concentration was different. Furthermore, there was a noticeable change in the equilibrium concentration in the midrib for all the positions. However, the values were irrelevant with respect to the order of midrib positions. For the midrib in positions 1 to 3, it took 91 h to reach equilibrium, and for the midrib in position 4, it took 115 h. This could be because the midrib becomes broader and thicker between positions 1 and 4, as discussed in Section 3.1 above.

**Figure 5:**
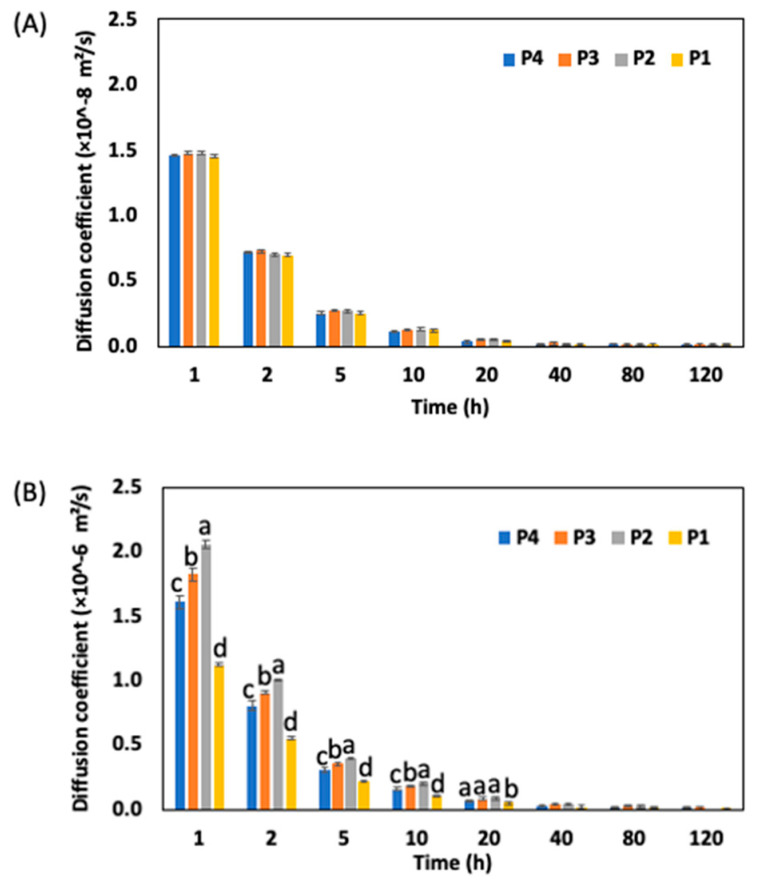
Diffusion coefficient for Chinese cabbage; (**A**) leaf blade and (**B**) midrib at position 4, 3, 2, and 1, respectively. Bars with different lower-case letters (a–d) represent measurement instances that are significantly different at *p* < 0.05.

The diffusion coefficient for the CC midrib and leaf blade is shown in Figure 5. The diffusion coefficient for the leaf blade was not significantly different based on the leaf positions at the analysis time intervals (Figure 5A). However, the midrib diffusion coefficient was significantly different until the 20 h soaking period and increased in the order of P1, P4, P3, and P2; for example, after the 1 h soaking period, the diffusion coefficients for the leaf midrib were 1.12, 1.61, 1.84, and 2.06 (× 10^−6^) for positions 1, 4, 3, and 2, respectively. The lower value at position 4 compared to position 2 and 3 may be because of the structural and morphological characteristics of the midrib. Although no study was found to have described the diffusion coefficient of vegetables and fruits based on their maturity, the study of Jun et al. [29] indicated that the fiber and dry matter content in the outer leaf of cabbage was higher than the inner leaf. Therefore, since the position is the outer one and has the leaf’s highest maturity, more fiber and dry matter may influence its ability to absorb soluble solids in the brine solution. Thus, the diffusion coefficient was lower at this point. Furthermore, the morphology of fruits and vegetables reveals a material that, rather than being isotropic, has a porous and very complex parenchymatic tissue. Barat et al., [30], for example, found that during the OD of apples, the microstructures of the apples contained an array of cells bounded by a tridimensional mesh of cell walls made of cellulose fibers, and most of the water and soluble solids inside the cells were in one or more large vacuoles. Thus, to further interpret the diffusion coefficient, the porosity of the CC midrib was measured. The porosity was 0.31 ± 0.01, 0.41 ± 0.01, 0.43 ± 0.01, and 0.47 ± 0.02 in the order of positions 1, 4, 3, and 2, respectively. This shows that positions 2 and 3 absorbed soluble solids faster than positions 1 and 4. Thus, the diffusion coefficient was highest in stem position 2. The variation in the porosity at all positions may also be accounted for by the presence of fiber, solid material, and the developmental stage, as the leaves in position 1 had a rigid structure and may have contained more fiber than in positions 2 and 3.

### 3.3. Shrinkage Analysis

**Figure 6:**
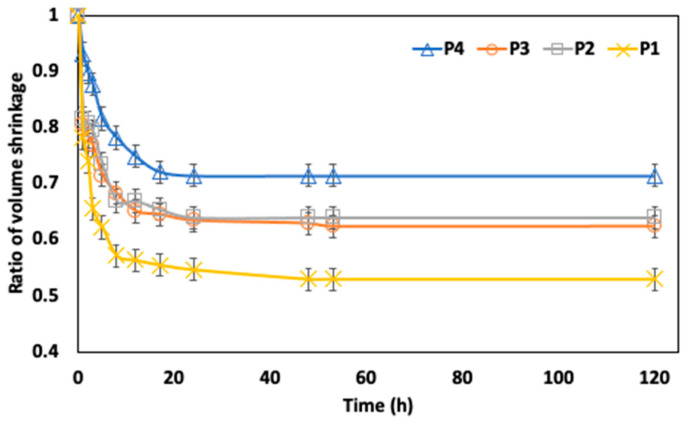
Shrinkage of Chinese cabbage midrib at different positions in brine solution of 2.0 mol/m^3^.

The ratio of shrinkage of the CC midrib during the brining process is presented in Figure 6. Shrinkage is a natural phenomenon induced by water loss during OD in foods and can be observed by the reduction in external dimensions (volume, surface area, length, etc.). A sharp decrease in the midrib volume was generally observed between 0 and 18 h of the brining process. This may be related to the high osmotic pressure generated by a highly concentrated solution, which may affect the plant capillary tissue and cause a rapid shrinking ratio. Generally, fruits and vegetables often have a high initial moisture content and experience substantial shrinkage throughout the dehydration process. However, as the brining process progresses, the rate of water loss slows, which causes shrinkage to slow down as well until the sample’s volume reaches a steady state [31]. This was the case with the CC midrib, as the shrinkage ratio was higher between 0 and 48 h and stabilized towards the later stages of the brining procedure, thereby showing that the volume of the midrib had not changed significantly.

### 3.4. Salt Concentration in Chinese Cabbage

**Figure 7:**
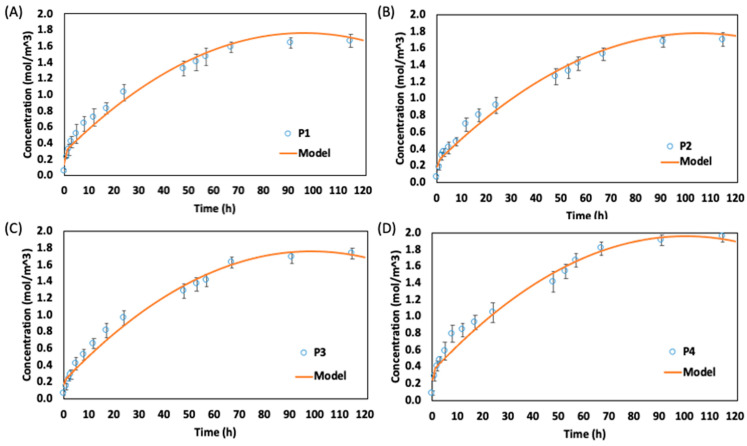
Numerical analysis and experimental results for soluble solid uptake by leaf blade in brine solution of 2.0 mol/m^3^: (**A**) position 1, (**B**) position 2, (**C**) position 3, and (**D**) position 4.

The diffusion of soluble solid content into the CC leaf blade during the brining process for the experimental analysis and numerical model (without shrinkage) is shown in Figure 7. The salt concentration increased at a rapid rate at the beginning of the brining process and began to slow down after about 10 h, thereby indicating a decrease in soluble solid uptake. The rapid uptake of soluble solids at the beginning is due to the large osmotic driving force between the fresh CC and the surrounding hypertonic medium. Several research groups have reported similar curves for the ODs of fruits and vegetables such as apples, pumpkins, carrots, and pears [32,33]. Subsequently, the concentration began to indicate a similar level after about 48 h. At this period, the NaCl concentrations inside the leaf blade were approaching equilibrium, and the diffusion rates had slowed. The fluctuation in the soluble solid concentration from about 80 to 120 h in the leaf blade of position 1 to 4 may be attributed to the migration of NaCl from the leaf blade to the midrib. This may occur because the leaf blade has reached its equilibrium and the midrib has not, and since they were initially not separated during the brining process, the soluble solid may migrate. The numerical analysis showed values within the experimental standard error range and had a low average relative error range of 1.18, 0.39, 0.34, and 1.38 for leaf blades of positions 4, 3, 2, and 1, respectively. This indicates that the model developed is suitable to predict the mass transfer characteristics of the CC leaf blade (Figure 7).

**Figure 8:**
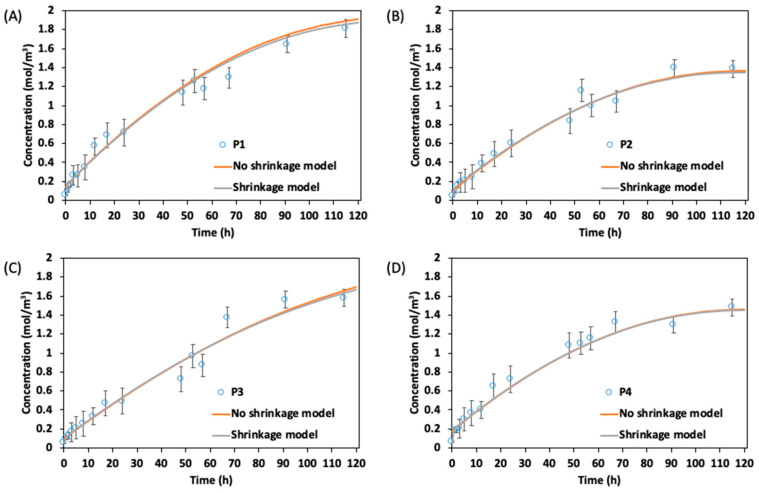
Numerical analysis (with and without shrinkage) and experimental results for soluble solid uptake by midrib in brine solution of 2.0 mol/m^3^: (**A**) position 1, (**B**) position 2, (**C**) position 3, and (**D**) position 4.

In the case of the CC midrib mass transfer analysis shown in Figure 8, the model with and without shrinkage was considered. The diffusion of the soluble solid content into the midrib during the brining process indicated similar rapid soluble solid uptake at the initial brining period as was found in the leaf blade. Generally, both numerical models (i.e., with and without shrinkage) indicated similar values between 0 and 48 h. After this period, the concentration became slightly different, especially in the midrib of position 1, but remained within the standard error range of the experimental result and with a low average relative error (i.e., 0.22, 0.12, 0.16, and 0.33 for positions 4, 3, 2, and 1, respectively). The changes in the soluble solid concentration after 48 h may be attributed to the macrostructural modifications that occur in the CC during OD because of the change in shape, structure, diffusivity, and the loss of moisture content.

**Figure 9:**
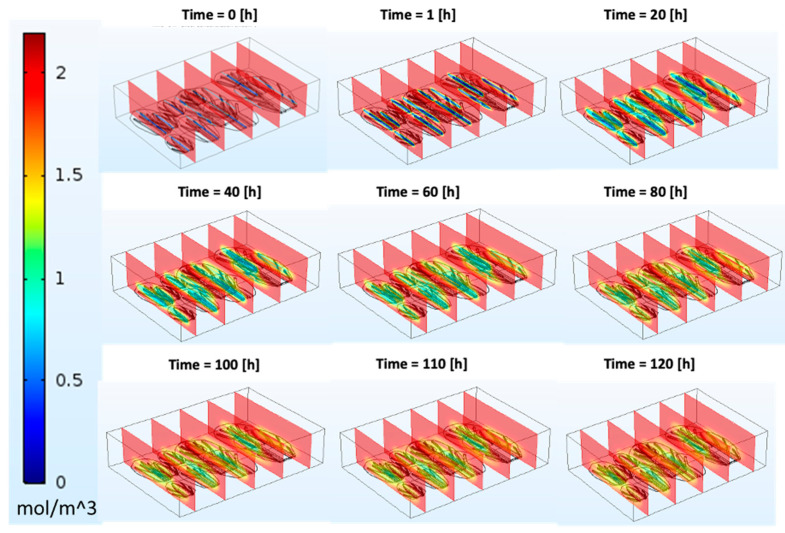
Contour description of soluble solid uptake during numerical analysis for leaf blade and midrib position of Chinese cabbage soaked in brine solution of 2.0 mol/m^3^.

The explanation for these macrostructural modifications during OD is complex. Salvatori and Alzamora [34] reported that at the beginning of the glucose osmotic process, the cell walls of apple tissues deformed and remained bound to the plasmalemma during cell shrinkage caused by water loss. However, after 200 min of osmosis, the original cell arrangements were relatively well preserved, and the cells appeared rounded like fresh ones. To a large extent, the cell walls recovered their original roundness and the intercellular spaces of their typical shape. The initial collapse of the tissue and the later recovery of the cell shape and intercellular areas would explain, at least partially, the fluctuations in the soluble concentration values observed for CC during OD. This is because we assumed that the CC during numerical analysis with the shrinkage model did not recover, as the shrinkage effect was applied from the start until the end of the brining process. Thus, the soluble solid concentration was lower compared to the model without shrinkage. Similarly, some other authors have also reported a cell recovery of fruit and vegetable tissues during OD, but only for a very long immersion, when compositional equilibrium between the concentrated solution and the fruit and vegetables had been reached [35,36]. Additionally, the contour image describing the migration of the soluble solid from the surrounding hypertonic solution into the CC leaves is shown in Figure 9. At the initial brining period, the leaf blade and midrib showed relatively low concentration values (<0.1 mol/m^3^), which increased with the brining period. A higher value was recorded in the leaf blade than at the midrib (Figure 9).

However, some authors, such as Zecchi and Gerla [16], have argued that not considering the shrinkage during the ODs in fruits and vegetables such as tomatoes had led to the overestimation of the diffusion coefficient that gave a significant difference in the soluble solid concertation. However, in our study for CC, we have found models with and without shrinkage to be within the relative error range of the experimental analysis. Based on this, the shrinkage model can be neglected during the analysis of OD for CC. This is because complex models usually require more computational power to solve analytical problems. For instance, in this study, the total time required to solve the OD process in CC without shrinkage was 48.35 min, whereas the model with shrinkage was solved in 74.52 min. Additionally, because of the continuous change in geometry for the shrinkage model, a continuous automatic remeshing had to be applied after the mesh quality had dropped below the set aspect ratio of 0.5. This implies that a higher computation power was used for a model with shrinkage. Therefore, to make the numerical analysis of the OD in CC simple and less expensive, the shrinkage model can be overlooked, and this can also be applied when simulating bigger CC structures such as half or whole vegetables.

## 4. Conclusions

The morphological properties of Chinese cabbage (CC) change were determined based on the level of leaf maturity, with leaf lengths ranging from 125 ± 4.7 to 290 ± 2.5 mm and midrib thickness ranging from 6.8 ± 0.2 to 7.5 ± 0.1 mm. The CC was divided into four sections using a dimensionless number. The identified CC leaf portions were analyzed for diffusion processes and the shrinkage effect in a NaCl solution with a concentration of 2.0 mol/m^3^ using the finite element method. Equilibrium concentrations of 1.744, 1.745, 1.744, and 1.807 mol/m^3^ were obtained for the CC leaf blade at positions 1, 2, 3, and 4, respectively, within approximately 56 h of brining time, while 1.632, 1.386, 1.579, and 1.480 mol/m^3^ were obtained for the CC midrib at positions 1, 2, 3, within approximately 91 h and at position 4 at approximately 115 h. A good quantitative agreement was obtained between the experimental and simulated results for the NaCl concentration in the CC leaf blade and midrib, with a low average relative error value between 0.1 and 1.4. Rapid soluble solid uptake was recorded for the CC leaf blade and midrib at the initial brining period between 0 and 18 h. This occurs due to high osmotic pressure between the cabbage and the brining solution and the presence of high initial moisture content that is lost from the CC to its hypertonic environment. The effect of shrinkage on the CC midrib during the brining process was significant within the first 18 h of the brining process. Similarly, the numerical analysis result comparing the shrinkage effect indicated that both models had similar soluble solid concentrations until about 48 h from the start of the process. This implies that, for a short-term brining process in CC, shrinkage might not have much effect on the soluble solid uptake and, thus, can be overlooked. However, between 48 and 120 h, the effect of shrinkage was visible, especially for the midrib at position 1; the changes in the concentration might be due to the recovery of the cell shape and the intracellular space in the CC cell structure. However, the NaCl concentrations predicted by the simulation models showed that the concentration from the shrinkage model was within an error range of the experimental values. Therefore, when modeling the diffusion process in the CC leaf blade and midrib, the shrinkage effect may be neglected to reduce the computational power used for simulation.

## Figures and Tables

**Table 1:** Measurement of the specifications of Chinese cabbage leaves at different positions.

		* Width (mm)						
		0%	25%		50%		80%	
Positions	Length (mm)	a	b	B	c	C	d	D
1	125 ± 4.7 ^d^	34 ± 2.2 ^d^	40 ± 1.5 ^c^	45 ± 2.5 ^c^	45 ± 2.5 ^c^	52.8 ± 1.9 ^d^	15.4 ± 1.1 ^a^	35 ± 2.3 ^d^
2	185 ± 4.6 ^c^	45.6 ± 2.3 ^c^	87.4 ± 2.3 ^a^	92.8 ± 3.4 ^b^	92.8 ± 3.4 ^b^	95.8 ± 2.7 ^c^	4.6 ± 1.1 ^c^	50.6 ± 2.1 ^c^
3	225 ± 6.2 ^b^	71.4 ± 1.8 ^a^	63.6 ± 5.8 ^b^	121.8 ± 5.1 ^a^	121.8 ± 3.4 ^a^	125.4 ± 2.7 ^b^	11.4 ± 1.1 ^b^	91.2 ± 1.7 ^b^
4	290 ± 2.5 ^a^	60.8 ± 1.3 ^b^	90.8 ± 3.6 ^a^	119.6 ± 5.8 ^a^	119.6 ± 5.8 ^a^	152.2 ± 3.7 ^a^	16.6 ± 1.1 ^a^	125.2 ± 3.1 ^a^

*—Width of Chinese cabbage at the location of measurement (i.e., 0%, 25%, 50%, and 80%). Values with different superscripts (a–d) in each column are significantly different at *p* < 0.05. The upper-case letters (B–D) represent the width of the leaf blade at each position, while the lower-case letters (a–d) represent the width of the midrib at each position.

**Table 2:** Measurement of the equilibrium concentration and required time to attain equilibrium in a Chinese cabbage leaf blade and midrib immersed in a NaCl concentration of 2.0 mol/m^3^.

	Position	Equilibrium Concentration (mol/m^3^)	Brining Time for Equilibrium Concentration (h)
Leaf blade	1	1.744	56
	2	1.745	56
	3	1.744	56
	4	1.807	56
Midrib	1	1.632	91
	2	1.386	91
	3	1.579	91
	4	1.480	115

## Data Availability

The data presented in this study are available upon request from the corresponding author. The data are not publicly available due to privacy restrictions.
